# Posttraumatic Stress Disorder and Type 2 Diabetes Outcomes in Veterans

**DOI:** 10.1001/jamanetworkopen.2024.27569

**Published:** 2024-08-13

**Authors:** Jeffrey F. Scherrer, Joanne Salas, Wenjin Wang, Kenneth E. Freedland, Patrick J. Lustman, Paula P. Schnurr, Beth E. Cohen, Allan S. Jaffe, Matthew J. Friedman

**Affiliations:** 1 Department of Family and Community Medicine, Saint Louis University School of Medicine, St Louis, Missouri; 2 Department of Psychiatry and Behavioral Neuroscience, Saint Louis University School of Medicine, St Louis, Missouri; 3 Advanced Health Data Research Institute, Saint Louis University School of Medicine, St Louis, Missouri; 4 Harry S. Truman Memorial Veterans’ Hospital, Columbia, Missouri; 5 Department of Psychiatry, Washington University School of Medicine, St Louis, Missouri; 6 National Center for PTSD, White River Junction, Vermont; 7 Department of Psychiatry, Geisel School of Medicine at Dartmouth, Hanover, New Hampshire; 8 Department of Medicine, University of California San Francisco School of Medicine, San Francisco; 9 San Francisco Veterans Affairs Medical Center, San Francisco, California; 10 Department of Cardiovascular Medicine, Mayo Clinic, Rochester, Minnesota; 11 Department of Laboratory Medicine and Pathology, Mayo Clinic, Rochester, Minnesota

## Abstract

**Importance:**

Posttraumatic stress disorder (PTSD) symptom reduction is linked with lower risk of incident type 2 diabetes (T2D), but little is known about the association between PTSD and comorbid T2D outcomes. Whether PTSD is a modifiable risk factor for adverse T2D outcomes is unknown.

**Objective:**

To determine whether patients with PTSD who improved and no longer met diagnostic criteria for PTSD had a lower risk of adverse T2D outcomes compared with patients with persistent PTSD.

**Design, Setting, and Participants:**

This retrospective cohort study used deidentified data from US Veterans Health Administration (VHA) historical medical records (from October 1, 2011, to September 30, 2022) to create a cohort of patients aged 18 to 80 years with comorbid PTSD and T2D. Data analysis was performed from March 1 to June 1, 2024.

**Exposures:**

Diagnoses of PTSD and T2D.

**Main Outcomes and Measures:**

The main outcomes were insulin initiation, poor glycemic control, any microvascular complication, and all-cause mortality. Improvement of PTSD was defined as no longer meeting PTSD diagnostic criteria, per a PTSD Checklist score of less than 33. Entropy balancing controlled for confounding. Survival and competing risk models estimated the association between meeting PTSD criteria and T2D outcomes. Subgroup analyses examined variation by age, sex, race, PTSD severity, and comorbid depression status.

**Results:**

The study cohort included 10 002 veterans. More than half of patients (65.3%) were aged older than 50 years and most (87.2%) were men. Patients identified as Black (31.6%), White (62.7%), or other race (5.7%). Before controlling for confounding with entropy balancing, patients who no longer met PTSD diagnostic criteria had similar incidence rates for starting insulin (22.4 vs 24.4 per 1000 person-years), poor glycemic control (137.1 vs 133.7 per 1000 person-years), any microvascular complication (108.4 vs 104.8 per 1000 person-years), and all-cause mortality (11.2 vs 11.0 per 1000 person-years) compared with patients with persistent PTSD. After controlling for confounding, no longer meeting PTSD criteria was associated with a lower risk of microvascular complications (hazard ratio [HR], 0.92 [95% CI, 0.85-0.99]). Among veterans aged 18 to 49 years, no longer meeting PTSD criteria was associated with a lower risk of insulin initiation (HR, 0.69 [95% CI, 0.53-0.88]) and all-cause mortality (HR, 0.39 [95% CI, 0.19-0.83]). Among patients without depression, no longer meeting PTSD criteria was associated with a lower risk of insulin initiation (HR, 0.73 [95% CI, 0.55-0.97]).

**Conclusions and Relevance:**

The findings of this cohort study of patients with comorbid PTSD and T2D suggest that PTSD is a modifiable risk factor associated with a modest reduction in microvascular complications. Further research is needed to determine whether findings are similar in non-VHA health care settings.

## Key Points

**Question:**

What is the association between meeting diagnostic criteria for posttraumatic stress disorder (PTSD) and risk of poor type 2 diabetes (T2D) outcomes?

**Findings:**

In this cohort study of 10 002 veterans, no longer meeting diagnostic criteria for PTSD was associated with a lower risk of microvascular complications. Among veterans aged 18 to 49 years, but not among those aged 50 to 80 years, no longer meeting PTSD criteria was associated with a lower likelihood of starting insulin and a lower risk of all-cause mortality.

**Meaning:**

The findings of this study suggest that PTSD is a modifiable risk factor for some adverse T2D outcomes among patients with comorbid PTSD and T2D.

## Teaser

This cohort study examines the association between meeting diagnostic criteria for posttraumatic stress disorder and risk of poor type 2 diabetes outcomes among a cohort of veterans.

## Introduction

Individuals with psychiatric disorders have a 20-year shorter life expectancy than those without mental illness, and despite advances in treatment, there has been little to no narrowing of the mental health disparity in mortality over the past 30 years.^1,2^ This disparity in mortality may be related to the association between mental illness and poor metabolic health. For example, posttraumatic stress disorder (PTSD) is associated with a significantly increased risk of incident T2D.^3,4,5,6,7,8,9,10^ Patients with comorbid PTSD and T2D have worse glycemic control, increased risk of hospitalization, and poorer self-reported health compared with patients with T2D alone.^11,12,13,14^

Some evidence suggests that PTSD might be a modifiable risk factor for T2D and adverse T2D outcomes. Treatment of PTSD and reduction in PTSD severity are linked to greater uptake of health-promoting behaviors such as smoking cessation and initiation of weight loss programs.^15,16,17^ Improvement of PTSD in comorbid psychiatric illness is associated with better overall well-being^18,19^ and with lower risk of some chronic health conditions, including T2D.^20^

To our knowledge, there is no existing literature on T2D outcomes after PTSD improvement among patients with comorbid PTSD and T2D. This study aimed to help fill this gap in the literature. If there is a reduced risk of adverse T2D outcomes among patients with PTSD who experience improvement and no longer meet PTSD criteria, then findings could be used to incentivize PTSD treatment and patients should be educated that improvement in PTSD may benefit comorbid T2D management. We investigated whether veterans with comorbid PTSD and T2D who no longer meet PTSD diagnostic criteria had a lower risk of starting insulin (a proxy for worsening T2D control), poor glycemic control, microvascular complications, or all-cause mortality compared with those with persistent PTSD. We planned subgroup analyses to determine whether age group, sex, race, PTSD severity, and comorbid depression status were significant effect modifiers.

## Methods

This cohort study used data from the US Veterans Health Administration (VHA). Because the data were deidentified and the investigators could not reidentify patients, the Saint Louis University and VHA institutional review boards deemed this study exempt and informed consent was waived. The study followed the Strengthening the Reporting of Observational Studies in Epidemiology (STROBE) reporting guideline.

### Study Population

All variables were created from deidentified VHA administrative medical record data from October 1, 2011, to September 30, 2022 (fiscal years FY2012-FY2022). Medical record data included diagnostic codes (*International Classification of Diseases, Ninth Revision* [*ICD-9*], and *Tenth Revision* [*ICD-10*]), *Current Procedural Terminology* codes, pharmacy records, laboratory results, vital signs, vital status, repeated PTSD Checklist (PCL) scores, and demographic information.

### Eligibility

A base sample of patients included those with at least 1 PTSD diagnosis (*ICD-9* code 309.81 or *ICD-10* code F43.1*) between FY2012 and FY2022. Potentially eligible patients had at least 1 PCL score (according to PTSD diagnostic criteria in the *Diagnostic and Statistical Manual of Mental Disorders, Fourth Edition* or *Fifth Edition* [PCL-4 or PCL-5]) from FY2013 to FY2019 that indicated probable PTSD, defined as a PCL score of 33 or greater (range, 0-80; higher scores indicate worse PTSD, lower scores indicate fewer symptoms) and at least 1 subsequent score that occurred at least 8 weeks and no more than 12 months after the first PCL score, to assess for symptom improvement in the exposure year.^21,22^

Following methods established by Moshier et al,^23^ PCL-4 scores were cross-walked to PCL-5 scores. To create an exposure year to measure PCL change and comorbid T2D, PCL scores of 33 or greater were measured between FY2013 and FY2019. Type 2 diabetes was defined with *ICD-9* (250.x0 and 250.x2) or *ICD-10* (E11*) codes. The end of this exposure year was the index (ie, baseline) date, which could occur between FY2014 and FY2020; this allowed for a 2-year look-back to measure covariates. This was a dynamic cohort, meaning that patients entered the study whenever they met the eligibility criteria. All eligible patients had a possible 2 to 9 years of follow-up.

**Figure 1.**
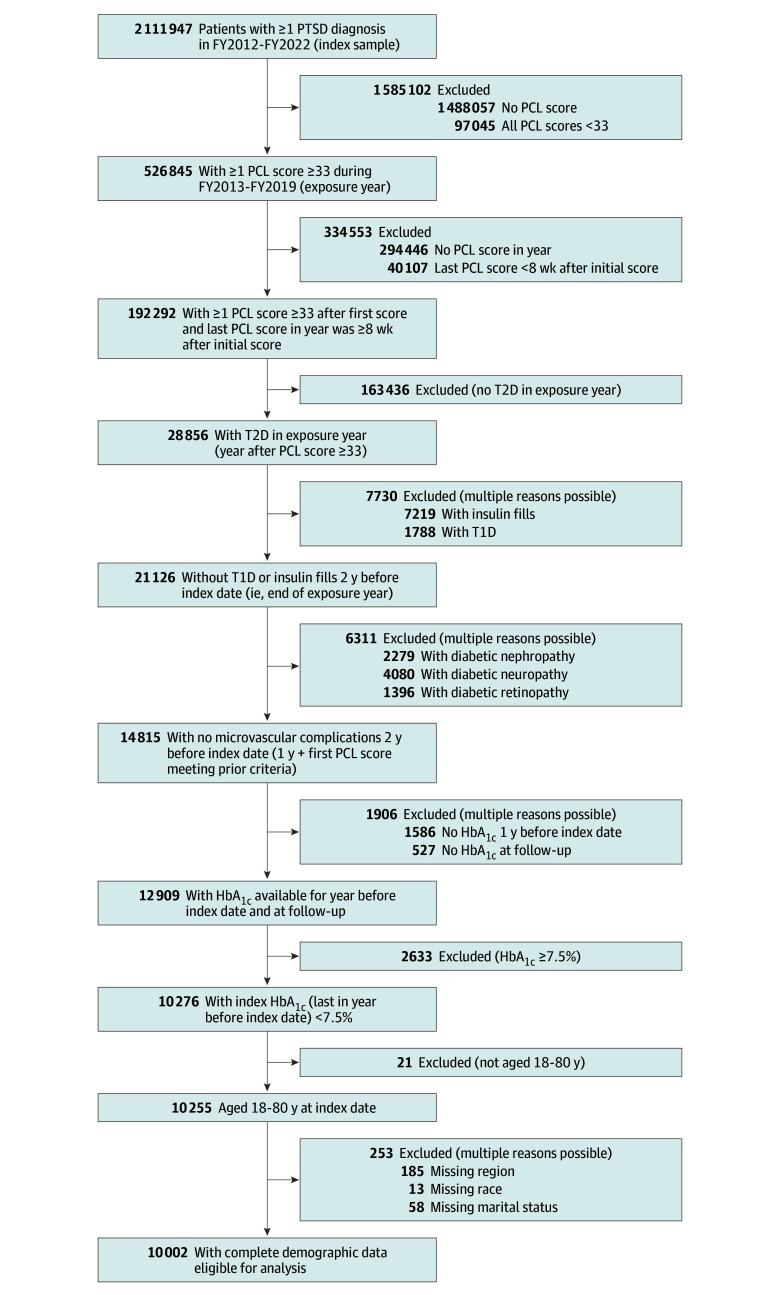
Cohort Eligibility FY indicates fiscal year; HbA_1c_, hemoglobin A_1c_; PCL, PTSD Checklist; PTSD, posttraumatic stress disorder; T1D, type 1 diabetes; T2D, type 2 diabetes.

**Figure 2.**
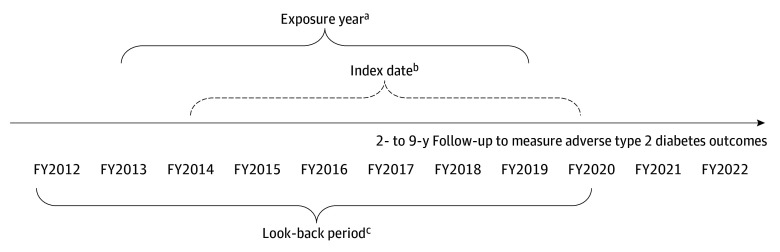
Dynamic Retrospective Cohort Study Design Patients entered the cohort when they met eligibility criteria. FY indicates fiscal year; PTSD, posttraumatic stress disorder. ^a^The exposure year could begin between 2013 and 2019 if the PTSD Checklist score was 33 or greater. ^b^The index date was the end of the exposure year, 2014 to 2020; patient did or did not meet PTSD criteria at the end of the exposure year. ^c^These years included a 2-year look-back to remove ineligible patients and to measure covariates.

The index date was the end of an exposure year that met the following criteria: (1) at least 1 PCL score 8 weeks after and within 12 months of the first PCL score of 33 or greater; (2) a T2D diagnosis coded in the exposure year; and (3) no insulin fills, type 1 diabetes, or microvascular complications in the 2 years before the end of the exposure year. After defining each patient’s index year, we then limited the sample to patients aged 18 to 80 years who had reasonably well-controlled hemoglobin A_1c_ (HbA_1c_) at index date (ie, last value in the year before index date <7.5%).^24^ Finally, those with missing demographic information were excluded, leaving an analytic sample of 10 002 patients diagnosed with PTSD who either met or no longer met the criteria for PTSD at the end of the exposure year. The sampling approach is illustrated in Figure 1. The study design is illustrated in Figure 2.

### Study Variables

Detailed variable definitions are presented in eTable 1 in Supplement 1. Type 2 diabetes outcomes were insulin initiation, poor glycemic control, any microvascular complication, and all-cause mortality. Insulin has traditionally been used as augmentation therapy, and we treated insulin initiation as a marker of poor T2D control. Recommendations on glycemic control vary and HbA_1c_ goals are based on patient age, comorbidities, presence of microvascular complications, and treatment preferences. Tight control with a goal HbA_1c_ of less than 7% is recommended by the American Diabetes Association and the VHA Department of Defense clinical practice guideline for many nonpregnant adults.^24,25^ However, for patients with limited life expectancy, increased risk of hypoglycemic complications, or both, HbA_1c_ goals are more liberal. Therefore, we used a cutoff HbA_1c_ of 7.5% or less to define good control, and we considered an HbA_1c_ greater than 7.5% during follow-up as poor glycemic control.^24,25^

Any microvascular complication was defined by the presence of *ICD-9* or *ICD-10* codes for diabetic nephropathy, retinopathy, or neuropathy. The VHA vital master file, which includes dates of death gathered from VHA medical records, the Social Security Administration, the Beneficiary Identification Records Locator Subsystem, and the Centers for Medicare & Medicaid Services, was used to measure all-cause mortality.

### Exposure

At the start of the exposure year, all patients had a PCL score of 33 or greater. Scores ranging from 31 to 33 are appropriate thresholds for identifying current PTSD.^22^ Patients were classified as no longer meeting PTSD criteria at index date if their last PCL score in the exposure year (using the aforementioned approach) was less than 33. Patients still met PTSD criteria if their PCL score remained at 33 or greater.

### Covariates

Unless otherwise indicated, covariates other than demographic factors were measured in the 2 years before index date. We controlled for index fiscal year and the following demographic characteristics: age, sex, patient-reported race as stored in the medical record (Black, White, or other [defined as American Indian or Alaska Native, Asian, or Native Hawaiian or Other Pacific Islander]), marital status, and geographic region. To account for detection bias, we controlled for high health care utilization in the 2 years before index date. High health care utilization was defined as the top 25th percentile of the distribution of average outpatient clinic visits per month per patient. We controlled for access to non–US Department of Veterans Affairs health insurance as a proxy for socioeconomic status.

Because the likelihood of a score falling below the PCL threshold of 33 partly depends on severity at index date, we controlled for very severe PTSD, which we defined as a PCL score of 66 or greater at the start of the exposure year. The following psychiatric comorbidities were controlled for: depression, dysthymia, any anxiety disorder (ie, social phobia, panic disorder, generalized anxiety disorder, or anxiety disorder not otherwise specified), obsessive-compulsive disorder, bipolar disorder, schizophrenia, alcohol or drug abuse or dependence, and smoking or nicotine dependence.

To investigate associations with PTSD improvement that could occur with or without treatment, we controlled for minimally adequate PTSD psychotherapy (at least 9 sessions in any 15-week period). We did so because patients who remain in treatment may be more likely to engage in other healthy behaviors that could reduce the risk of T2D complications compared with those who do not. We controlled for other psychotropic therapies, including 12 or more weeks of antidepressant therapy (ie, at least acute-phase treatment). Given their metabolic side effects, we controlled for sustained use of atypical antipsychotics, defined as at least 2 fills in any 6-month period per prior research.^26,27,28^

Physical comorbidities included metabolic and cardiovascular conditions posited to be more common in PTSD and to contribute to worse T2D outcomes. These conditions included obesity, hyperlipidemia, atrial fibrillation, angina, heart failure, left ventricular hypertrophy, myocardial infarction, peripheral vascular disease, and stroke. We controlled for HbA_1c_ at index date, which was the last HbA_1c_ measurement available in the year before index date. Finally, we controlled for sustained use of antidiabetic medications (metformin, sulfonylurea, dipeptidyl peptidase 4 inhibitors, glucagon-like peptide 1 agonists, sodium-glucose cotransporter 2 inhibitors, and thiazolidinediones).

### Statistical Analysis

#### Control for Confounding

We controlled for confounding with entropy balancing to remove differences in the distribution of covariates between patients whose PTSD persisted vs those who no longer met PTSD criteria.^29,30^ In this way, balancing emulates randomization. Entropy balancing weights were computed using the WeightIt package in R, version 4.2.1 (R Project for Statistical Computing).^31^ The percentage of standardized mean difference (SMD% = 100 × SMD) was computed to evaluate balance. Well-balanced covariates have an SMD percentage of less than 10%.^32^ This balancing approach to mitigate confounding permits analysis of the total average treatment effect of no longer meeting PTSD criteria on risk of adverse T2D outcomes.

Bivariate comparisons between covariates and whether patients continued to meet PTSD criteria were evaluated using χ^2^ tests or independent samples *t* tests. We used SMD percentages to measure balance before and after entropy balancing. Competing risk survival models,^33^ which account for the competing risk of death, were used to estimate the associations between no longer meeting PTSD criteria and each T2D outcome. A Cox proportional hazards regression model was computed to estimate risk of all-cause mortality. The censor date for starting insulin and for microvascular complications was either death or last visit date in follow-up; for poor glycemic control, it was death or last HbA_1c_ measurement. The censor date for the all-cause mortality model was the end of follow-up (September 30, 2022). Follow-up time was calculated as months from index date to either the outcome date or the censor date. All models were fitted before and after entropy balance weighting to calculate hazard ratios (HRs) and 95% CIs. Weighted models used robust, sandwich-type variance estimators for CIs.^32^ The proportional hazards assumption was tested and met for all models (*P* > .10).

#### Subgroup Comparisons

In previous studies, younger patients (18-50 vs >50 years) tended to have worse glycemic control,^34^ Black individuals tended to have more diabetes complications than White patients,^34^ and T2D distress was more common among female individuals compared with male indviduals.^35^ Comorbid depression has also been associated with poor T2D outcomes.^36^ Finally, more severe PTSD is linked to increased risk of microvascular complications.^37^ Therefore, planned subgroup analyses were performed for age group (18-49 vs 50-80 years), race (Black, White, or other), sex (male or female), comorbid depression status, and PTSD severity at the start of the exposure year (PCL score indicating severe PTSD: ≥66 vs <66). Entropy balancing was computed for each subgroup analysis. An interaction term for whether patients continued to meet PTSD criteria and each stratification variable assessed whether effect modification was present (*P* < .05 indicated significant modification).

#### Post Hoc Sensitivity Analysis

It is possible that a PCL score of less than 33 is not sufficient improvement to result in a change in physiology or in health behaviors associated with better T2D outcomes. To explore this issue, we conducted post hoc analyses using additional PCL score thresholds for change in PTSD severity. We compared risk of T2D outcomes among patients with PCL scores of 18 or less (reference group), 33 to 65, or 66 to 80. Separate sensitivity analyses modeled poor glycemic control as an HbA_1c_ greater than 7.0%. Finally, we adjusted for use of the VHA’s evidence-based weight loss program MOVE!^38^ because PTSD improvement may be linked to adopting healthier behavior and fewer adverse T2D outcomes.

Data analysis was performed from March 1 to June 1, 2024. The main statistical analyses were performed using SAS, version 9.4 (SAS Institute), with α = .05 (2-tailed).

## Results

This study included 10 002 veterans. Of these patients, 65.3% were aged older than 50 years, 61.4% were married, 87.2% were men, and 12.8% were women (Table 1). Patients identified as Black (31.6%), White (62.7%), or other race (5.7%). A total of 19.2% of patients had severe PTSD; 20.2% no longer met PTSD criteria by the end of an exposure year. More than half of patients had comorbid depression (66.6%) and most had obesity (70.3%). During follow-up, 11.6% of patients started insulin, 43.7% had an HbA_1c_ greater than 7.5%, 39.4% had any microvascular complication, and 5.8% died.

**Table 1.** Baseline Characteristics of Patients With Comorbid PTSD and Type 2 Diabetes by PTSD Status^a^

Characteristic	All patients (N = 10 002)	Patient PTSD status		SMD, %	
		Met criteria (n = 7878)	No longer met criteria (n = 2124)	Unweighted	Weighted
Index fiscal year					
2014	1919 (19.2)	1556 (19.8)	363 (17.1)	6.9	0
2015	1231 (12.3)	967 (12.3)	264 (12.4)	0.5	0
2016	915 (9.1)	711 (9.0)	204 (9.6)	2.0	0.01
2017	865 (8.6)	717 (9.1)	148 (7.0)	7.9	0.01
2018	1388 (13.9)	1097 (13.9)	291 (13.7)	0.7	0
2019	1797 (18.0)	1418 (18.0)	379 (17.8)	0.4	0.01
2020	1887 (18.9)	1412 (17.9)	475 (22.4)	11.1	0.01
Age, y					
18-39	1203 (12.0)	985 (12.5)	218 (10.3)	7.1	0.01
40-49	2263 (22.6)	1868 (23.7)	395 (18.6)	12.6	0
50-59	2732 (27.3)	2189 (27.8)	543 (25.6)	5.0	0
≥60	3804 (38.0)	2836 (36.0)	968 (45.6)	19.6	0.01
Sex					
Male	8717 (87.2)	6897 (87.6)	1820 (85.7)	5.5	0
Female	1285 (12.8)	981 (12.4)	304 (14.3)	5.5	0
Race					
Black	3162 (31.6)	2594 (32.9)	568 (26.7)	13.6	0.01
White	6267 (62.7)	4812 (61.1)	1455 (68.5)	15.6	0
Other^b^	573 (5.7)	472 (6.0)	101 (4.8)	5.5	0
Marital status, married	6139 (61.4)	4799 (60.9)	1340 (63.1)	4.5	0.01
US region					
Northeast	1004 (10.0)	756 (9.6)	248 (11.7)	6.8	0.01
Midwest (North Central)	1838 (18.4)	1404 (17.8)	434 (20.4)	6.6	0.02
South	5289 (52.9)	4273 (54.2)	1016 (47.8)	12.8	0.01
West	1871 (18.7)	1445 (18.3)	426 (20.1)	4.4	0
VA-only insurance	3972 (39.7)	3219 (40.9)	753 (35.5)	11.2	0
High health care utilization	2500 (25.0)	1993 (25.3)	507 (23.9)	3.3	0
HbA_1c_, mean (SD), %	6.3 (0.6)	6.3 (0.6)	6.3 (0.6)	2.0	0
Severe PTSD^c^	1924 (19.2)	1748 (22.2)	176 (8.3)	39.4	0
Psychiatric comorbidity					
Depression	6656 (66.6)	5341 (67.8)	1315 (61.9)	12.4	0
Dysthymia	483 (4.8)	383 (4.9)	100 (4.7)	0.7	0
Anxiety	3205 (32.0)	2552 (32.4)	653 (30.7)	3.6	0
Obsessive compulsive disorder	104 (1.0)	84 (1.1)	20 (0.9)	1.3	0.02
Bipolar disorder	866 (8.7)	682 (8.7)	184 (8.7)	0.02	0.01
Schizophrenia	237 (2.4)	189 (2.4)	48 (2.3)	0.9	0.01
Substance abuse or dependence					
Alcohol	2783 (27.8)	2233 (28.3)	550 (25.9)	5.5	0.01
Drugs	1677 (16.8)	1341 (17.0)	336 (15.8)	3.3	0.01
Smoking or nicotine	4544 (45.4)	3634 (46.1)	910 (42.8)	6.6	0.01
PTSD treatment					
Adequate psychotherapy	3104 (31.0)	2367 (30.1)	737 (34.7)	10.0	0
Antidepressant medication for ≥12 wk	7895 (78.9)	6277 (79.7)	1618 (76.2)	8.5	0.01
Atypical antipsychotic use	2137 (21.4)	1775 (22.5)	362 (17.0)	13.8	0
Physical comorbidity					
Obesity	7031 (70.3)	5591 (71.0)	1440 (67.8)	6.9	0.01
Hyperlipidemia	7622 (76.2)	5953 (75.6)	1669 (78.6)	7.2	0
Atrial fibrillation	401 (4.0)	310 (3.9)	91 (4.3)	1.8	0.01
Angina	120 (1.2)	99 (1.3)	21 (1.0)	2.5	0.0.6
Congestive heart failure	402 (4.0)	322 (4.1)	80 (3.8)	1.7	0
Hypertension	7382 (73.8)	5768 (73.2)	1614 (76.0)	6.4	0.01
Left ventricular hypertrophy	253 (2.5)	194 (2.5)	59 (2.8)	2.0	0.04
Myocardial infarction	132 (1.3)	98 (1.2)	34 (1.6)	3.0	0.04
Peripheral vascular disease	341 (3.4)	255 (3.2)	86 (4.1)	4.3	0.01
Stroke	279 (2.8)	219 (2.8)	60 (2.8)	0.3	0.02
Antidiabetic medication use					
Metformin	4966 (49.6)	3853 (48.9)	1113 (52.4)	7.0	0
Sulfonylurea	1223 (12.2)	941 (11.9)	282 (13.3)	4.0	0.01
DPP-4 inhibitor	167 (1.7)	117 (1.5)	50 (2.4)	6.3	0.01
GLP-1 agonist	77 (0.8)	50 (0.6)	27 (1.3)	6.6	0.01
SGLT-2 inhibitor	57 (0.6)	39 (0.5)	18 (0.9)	4.3	0.02
Thiazolidinedione	85 (0.9)	69 (0.9)	16 (0.8)	1.4	0.03

Abbreviations: DPP-4, dipeptidyl peptidase 4; GLP-1, glucagon-like peptide 1; HbA_1c_, hemoglobin A_1c_; PCL, PTSD Checklist; PTSD, posttraumatic stress disorder; SGLT-2, sodium-glucose cotransporter 2; SMD, standardized mean difference; VA, US Department of Veterans Affairs.

^a^ Unless indicated otherwise, values are presented as the No. (%) of patients.

^b^ Defined as American Indian or Alaska Native, Asian, or Native Hawaiian or Other Pacific Islander.

^c^ First PCL score of 66 or greater.

The median time between the first PCL score obtained in the exposure year to the last PCL score (index PCL score) was 29 (IQR, 17-42) weeks. Among patients with persistent PTSD, the median time between first and last scores was 29 (IQR, 17-43) weeks. Among patients who no longer met criteria for PTSD, the median time between first and last scores was 26 (IQR, 15-40) weeks.

Table 1 presents the covariate distributions, before balancing, among patients who did vs did not continue to meet PTSD criteria. Patients who no longer met PTSD criteria were more likely to be aged older than 60 years (SMD, 19.6%). Severe PTSD (SMD, 39.4%) and atypical antipsychotic use (SMD, 13.8%) were more prevalent among patients who continued to meet PTSD criteria. After weighting the data, all covariates balanced between those who did and did not meet PTSD criteria (weighted SMD < 10%).

Crude incidence rates per 1000 person-years for insulin initiation, poor glycemic control, any microvascular complication, and all-cause mortality are presented in Table 2. Incidence rates did not differ substantially between patients who no longer met PTSD criteria compared with patients with persistent PTSD. Before controlling for confounding with entropy balancing, patients who no longer met PTSD criteria had similar incidence rates for starting insulin (22.4 vs 24.4 per 1000 person-years), poor glycemic control (137.1 vs 133.7 per 1000 person-years), any microvascular complication (108.4 vs 104.8 per 1000 person-years), and all-cause mortality (11.2 vs 11.0 per 1000 person-years) compared with those with persistent PTSD.

**Table 2.** Type 2 Diabetes Outcomes: Cumulative Incidence Percent and Incidence Rate by PTSD Status

Outcome	PTSD status		*P* value
	Met criteria (n = 7878)	No longer met criteria (n = 2124)	
Starting insulin			
No. (%) of events	917 (11.6)	219 (10.3)	.09
Incidence rate per 1000 person-years	24.4	22.4	.25
Poor glycemic control			
No. (%) of events	3442 (43.7)	903 (42.5)	.33
Incidence rate per 1000 person-years	133.7	137.1	.50
Any microvascular complication			
No. (%) of events	3107 (39.4)	833 (39.2)	.85
Incidence rate per 1000 person-years	104.8	108.4	.39
Mortality			
No. (%) of events	455 (5.8)	120 (5.7)	.83
Incidence rate per 1000 person-years	11.0	11.2	.85

Abbreviation: PTSD, posttraumatic stress disorder.

Both before and after controlling for confounding, the incidence of starting insulin, poor glycemic control, or mortality did not significantly differ between patients who no longer met criteria for PTSD compared with those with persistent PTSD (Table 3). After controlling for confounding using weighted data, no longer meeting criteria for PTSD was associated with a significantly lower risk of microvascular complications (hazard ratio, 0.92 [95% CI, 0.85-0.99]).

**Table 3.** Association Between PTSD Status and Type 2 Diabetes Outcomes Before and After Entropy Balance Weighting to Control for Confounding

Outcome	PTSD status	
	Meets PTSD criteria [reference]	No longer meets PTSD criteria, HR (95% CI)
Starting insulin^a^		
Unweighted	1 [Reference]	0.92 (0.79-1.06)
Weighted	1 [Reference]	0.93 (0.80-1.07)
Poor glycemic control^a^		
Unweighted	1 [Reference]	1.01 (0.94-1.08)
Weighted	1 [Reference]	1.00 (0.93-1.08)
Any microvascular complication^a^		
Unweighted	1 [Reference]	1.03 (0.95-1.11)
Weighted	1 [Reference]	0.92 (0.85-0.99)
Mortality^b^		
Unweighted	1 [Reference]	1.03 (0.85-1.26)
Weighted	1 [Reference]	0.99 (0.79-1.24)

Abbreviations: HR, hazard ratio; PTSD, posttraumatic stress disorder.

^a^ Competing risk survival models.

^b^ Cox proportional hazards regression model.

Subgroup analyses revealed statistically significant interactions by age group and meeting PTSD criteria and risk of insulin initiation and all-cause mortality (eTable 2 in Supplement 1). Among patients aged 18 to 49 years, but not among those aged 50 to 80 years, no longer meeting PTSD criteria was associated with a lower likelihood of starting insulin (HR, 0.69 [95% CI, 0.53-0.88]; *P* = .003) and a lower risk of all-cause mortality (HR, 0.39 [95% CI, 0.19-0.83]; *P* = .008).

The association between no longer meeting PTSD criteria and T2D outcomes did not significantly differ by sex (eTable 3 in Supplement 1), by race (eTable 4 in Supplement 1), or by severe PTSD (eTable 6 in Supplement 1) at index date. However, there was a significant interaction between depression and no longer meeting PTSD criteria and risk of starting insulin. Among those without depression, no longer meeting PTSD criteria was associated with a lower risk of insulin initiation (HR, 0.73 [95% CI, 0.55-0.97]; *P* = .03) (eTable 5 in Supplement 1).

Sensitivity analyses (eTable 7 in Supplement 1) using different PCL thresholds at index date to define exposure groups revealed that compared with a PCL score of 18 or less at index date, the risk of microvascular complications was significantly greater for those with a PCL score of 19 to 32 (HR, 1.23 [95% CI, 1.06-1.42]) and nearly identical point estimates were observed for PCL scores of 33 to 65 and 66 to 80. Thus, there appeared to be a greater magnitude of reduced risk of microvascular complications among those with PCL scores of 18 or less. There were no associations between these other PCL thresholds and insulin initiation, glycemic control, and all-cause mortality.

Results did not change in sensitivity analyses treating poor glycemic control as an HbA_1c_ greater than 7.0%. Finally, results did not change after adjusting for use of the MOVE! program.

## Discussion

In this cohort study of VHA patients with comorbid PTSD and T2D, we observed that no longer meeting diagnostic criteria for PTSD was associated with a modest (8%) reduction in risk of microvascular complications, compared with continuing to meet PTSD criteria. Among patients aged 18 to 49 years (but not among older patients), no longer meeting PTSD criteria was associated with a lower risk of insulin initiation and all-cause mortality. It is logical that younger, adult VHA patients would have a reduced risk of insulin initiation and mortality because the accumulation of comorbid conditions that contribute to poor health outcomes increases with aging and likely limits the ability to detect associations between PTSD improvement and T2D outcomes in those aged older than 50 years.

Michopoulos et al^39^ described PTSD as a metabolic disease with common underlying mechanisms related to the inflammatory response. From this perspective, it is possible that physiological abnormalities in the hypothalamic-pituitary-adrenal axis, changes in metabolic hormones, and poor diet and lack of exercise explain why PTSD is a risk factor for prediabetes and T2D. Although associated with incident T2D, the present findings, along with sparse existing literature,^40^ suggest that large decreases in PTSD severity may have a modest favorable association with some T2D outcomes, particularly microvascular complications. In younger age groups and among those without comorbid depression, the magnitude of associations was much greater. Clinicians managing T2D among patients with PTSD should ensure that evidence-based treatments are used to reduce PTSD symptoms and improve T2D outcomes, particularly among younger individuals.

We observed that patients without depression and no longer meeting PTSD criteria were statistically significantly less likely to start insulin. This finding is consistent with prior observations that PTSD is associated with poor glycemic control.^11,12,13^ In addition, this finding is aligned with the link between depression and hyperglycemia^41^ and is consistent with evidence that depression and correlated inflammation and oxidative stress increase risk of poor T2D outcomes.^36,42^ This finding could also be related to poor diabetes self-management and poor medication adherence among persons with PTSD and depression.^42^

In a 2023 study of PTSD severity in patients with comorbid T2D, we observed that severe PTSD, compared with mild PTSD, was associated with an increased risk of microvascular complications but not with poor glycemic control and insulin initiation.^37^ The lack of associations between PTSD status and glycemic control agrees with a prior study demonstrating that HbA_1c_ values in comorbid PTSD and T2D do not meaningfully differ between groups with both PTSD and depression, with PTSD alone, with depression alone, and without PTSD and depression.^43^ Another explanation for good glycemic control is more frequent glucose self-monitoring, which has been observed in individuals with more severe PTSD.^44^ The lack of association between PTSD status and glycemic control might be related to the VHA’s excellent T2D management relative to the private sector.^45^ The VHA outperforms the civilian sector in T2D treatment, with a much higher percentage of VHA patients achieving control than the private sector.^45^ It is possible the increases in insulin use that we observed in some subgroups reflect intensification of medications that prevented patients from crossing thresholds for poor control. With such well-managed T2D, we may have lacked sufficient variability in HbA_1c_ values in follow-up to detect an association with PTSD improvement.

### Limitations

This study had some limitations. We do not know how long the patients in this cohort had comorbid PTSD and T2D. Heterogeneity in duration of these conditions could bias our results. We could not overcome this limitation because there were too few eligible patients to form a cohort of patients with new-onset PTSD and newly diagnosed T2D. Yet such a cohort would identify an atypical pattern of onset that would not represent the majority of patients with comorbid PTSD and T2D. This study is based on medical record data from the VHA and results may not generalize to non-VHA health systems. Additional research in more diverse samples is needed. Misclassification is a risk in retrospective cohort designs, particularly when relying on historical medical record data. For example, we might have biased analyses toward the null if we substantially misclassified patients as unaffected if they had undiagnosed microvascular complications. We controlled for a large number of potential confounders but we are not able to rule out unmeasured confounding. The VHA’s superior diabetes management may have reduced our ability to detect adverse T2D outcomes. The majority of the sample was male and results may not generalize to female individuals with PTSD and T2D. Finally, although a PCL cut point of 33 is a good indicator of a PTSD diagnosis,^22^ it is important to remember that a questionnaire does not provide a definitive diagnosis of PTSD.

## Conclusions

Among patients with comorbid PTSD and T2D in this cohort study, no longer meeting PTSD diagnostic criteria was associated with a modest reduction in risk of microvascular complications. Overall, our results suggest that PTSD is a modifiable risk factor for some adverse T2D outcomes. Further research is needed to determine whether findings are similar in non-VHA health care settings.

## Notes

Supplement 1. **eTable 1.** Variable Definitions **eTable 2.** Age Subgroup Analyses **eTable 3.** Gender Subgroup Analyses **eTable 4.** Race Subgroup Analyses **eTable 5.** Depression Subgroup Analyses **eTable 6.** Posttraumatic Stress Disorder (PTSD) Severity Subgroup Analyses **eTable 7.** Four Levels of PTSD Checklist (PCL) Severity Groups

Supplement 2.
Data Sharing Statement
