# Supplementary material for: Posttraumatic Stress Disorder and Type 2 Diabetes Outcomes in Veterans
Source: JAMA Netw Open. 2024 Aug 13;7(8):e2427569. doi: 10.1001/jamanetworkopen.2024.27569 (PMC11322846; doi:10.1001/jamanetworkopen.2024.27569)
Supplement: Supplement 1. — eTable 1. Variable Definitions eTable 2. Age Subgroup Analyses eTable 3. Gender Subgroup Analyses eTable 4. Race Subgroup Analyses eTable 5. Depression Subgroup Analyses eTable 6. Posttraumatic Stress Disorder (PTSD) Severity Subgroup Analyses eTable 7. Four Levels of PTSD Checklist (PCL) Severity Groups [file jamanetwopen-e2427569-s001.pdf]

Supplementary Online Content

Scherrer JF, Salas J, Wang W, et al. Posttraumatic stress disorder and type 2 diabetes outcomes in veterans. *JAMA Netw Open*. 2024;7(8):e2427569. doi:10.1001/jamanetworkopen.2024.27569

eTable 1. Variable Definitions

eTable 2. Age Subgroup Analyses

eTable 3. Gender Subgroup Analyses

eTable 4. Race Subgroup Analyses

eTable 5. Depression Subgroup Analyses

eTable 6. Posttraumatic Stress Disorder (PTSD) Severity Subgroup Analyses

eTable 7. Four Levels of PTSD Checklist (PCL) Severity Groups

This supplementary material has been provided by the authors to give readers additional information about their work.

© 2024 Scherrer JF et al. *JAMA Network Open.*

<table>
<tr><td colspan="2">eTable 1. Variable Definitions</td></tr>
<tr><td>Variable</td><td>Definition</td></tr>
<tr><td>Index/baseline</td><td>PCL scores indicate PCL-4 or PCL-5 scores. PCL-4 scores were cross walked to PCL-5.

Exposure year (the start can occur from FY13 to FY19): Year containing a PCL≥33 with a second score at least 8 weeks after. The end of the exposure year is index as defined below. Index date can occur from FY14 to FY20.

Index date = end of the first occurring exposure year where: a) PCL score ≥33; b) a second PCL available within a year that is at least 8 weeks after; c) T2DM in exposure year; d) No T1DM or insulin fills for 2-years prior to index; and d) no microvascular complications in 2-years prior</td></tr>
<tr><td>Follow-up time</td><td>Months from index to study end date (incident outcome or censoring).
Censoring for each outcome:
Time to insulin start – death or last visit
Time to microvascular complication – death or last visit
Time to poor glycemic control – death or last A1c measurement
All-cause mortality – 9/30/22</td></tr>
<tr><td>T2DM</td><td>ICD-9 code: 250.x0, 250.x2;
ICD-10 code: E11.x</td></tr>
<tr><td>T1DM</td><td>ICD-9 code: 250.x1, 250.x3;
ICD-10 code: E10.x</td></tr>
<tr><td colspan="2">Outcomes</td></tr>
<tr><td>Starting insulin</td><td>First insulin fill in follow-up</td></tr>
<tr><td>Poor glycemic control</td><td>First A1c in follow-up ≥7.5</td></tr>
<tr><td>Any microvascular complication</td><td>Composite from list below:
<table>
<tr><td>Microvascular complication</td><td>ICD9</td><td>ICD10</td></tr>
<tr><td>Diabetic retinopathy</td><td>250.50, 250.52, 362.0x, 366.41</td><td>E11.3x, E13.3x, E14.3x, H28.0, H36.0</td></tr>
<tr><td>Diabetic neuropathy</td><td>250.60, 250.62, 357.2</td><td>E11.4x, E13.4x, E14.4x, G63.2, G59.0</td></tr>
<tr><td>Diabetic nephropathy</td><td>250.40, 250.42, 585.x</td><td>E11.2x, E13.2x, E14.2x, N08.3, N18.x</td></tr>
</table></td></tr>
<tr><td>Mortality</td><td>All-cause mortality from VHA vital status file</td></tr>
<tr><td colspan="2">Exposures</td></tr>
<tr><td>PCL – Meeting PTSD criteria</td><td>Last PCL in the exposure year is ≥33 = persistent PTSD and PCL<33 indicates no longer meets PTSD criteria.</td></tr>
</table>

© 2024 Scherrer JF et al. *JAMA Network Open.*

Comorbidity covariates – measured in 2-years prior to index (end of exposure year). At least one occurrence/diagnostic code unless otherwise specified.	
PTSD severe	If initial PCL in exposure year is ≥66
Depression	ICD-9 code: 296.2x, 296.3x, 311;<br>ICD-10 code: F32.0-F32.5, F32.9, F33.0-F33.3, F33.4x, F33.9<br>- 2 outpatient occurrences (on different days) in same 12-month period or 1 inpatient occurrence
Dysthymia	ICD-9 code: 300.4;<br>ICD-10 code: F34.1<br>- 2 outpatient occurrences (on different days) in same 12-month period or 1 inpatient occurrence
Anxiety	ICD-9 code: 300.00, 300.01, 300.02, 300.23;<br>ICD-10 code: F40.1x, F41.0, F41.1, F41.9<br>- Composite of panic disorder, anxiety disorder not otherwise specified, social phobia, and generalized anxiety disorder<br>- 2 outpatient occurrences (on different days) in same 12-month period or 1 inpatient occurrence
Obsessive compulsive disorder	ICD-9 code: 300.3;<br>ICD-10 code: F42<br>- 2 outpatient occurrences (on different days) in same 12-month period or 1 inpatient occurrence
Schizophrenia	ICD-9 code: 295.x;<br>ICD-10 code: F20.x, F25.x
Bipolar disorder	ICD-9 code: 296.0x, 296.1x, 296.4x-296.8x;<br>ICD-10 code: F30.x, F31.x
Alcohol abuse/dependence	ICD-9 code: 303.9x, 305.0x;<br>ICD-10 code: F10.x
Any drug abuse/dependence	ICD-9 code: 304.0x, 304.1x, 304.2x, 304.3x, 304.4x, 304.5x, 304.6x, 304.7x, 304.8x, 304.9x, 305.2x, 305.3x, 305.4x, 305.5x, 305.6x, 305.7x, 305.9x<br>ICD-10 code: F11.x, F12.x, F13.x, F14.x, F15.x, F16.x, F18.x, F19.x<br>- Composite of sedative, cocaine, cannabis, amphetamine, hallucinogens, 'other,' opioid, opioid with other SUD, other SUD excluding opioid, unspecified drug abuse/dependence.
Smoking/nicotine dependence	ICD-9 code: V15.82, 305.1;<br>ICD-10 code: Z87.891, Z72.0, F17.20x, F17.21x<br>Present in health factor data as current smoker
Adequate PTSD treatment	At least 9 unique clinic stops (clinic stop codes: 516, 540, 541, 561, 562) for PTSD psychotherapy in any 15-week period.
Adequate ADM treatment	At least 12 weeks of continuous use. Continuous use is defined as no gaps > 30 days in fills. All SSRI, SNRI, MAOI, TCA, and other types.
Atypical antipsychotic use	Sustained use = is at least 2 fills in any 6-month period. Atypical antipsychotics: aripiprazole, asenapine, brexipiprazole, cariprazine, clozapine, iloperidone, lurasidone, olanzapine, paliperidone, pimavanserin, quetiapine, risperidone, ziprasidone

© 2024 Scherrer JF et al. *JAMA Network Open.*

Obesity	Last BMI on/before index is ≥ 30
Hyperlipidemia	ICD-9 code: 272.0 – 272.4;<br>ICD-10 code: E78.0x, E78.1, E78.2, E78.3, E78.4x, E78.5, E78.8x, E78.9
Atrial Fibrillation	ICD-9 code: 427.3x;<br>ICD-10 code: I48.x
Angina	ICD-9 code: 411.81, 411.89, 413.0;<br>ICD-10 code: I20.0, I24.8, I24.9, I25.110, I25.700, I25.710, I25.720, I25.730, I25.750, I25.760, I25.790
Congestive heart failure	ICD-9 code: 398.91, 402.01, 402.11, 402.91, 404.01, 404.03, 404.11, 404.13, 404.91, 404.93, 428.x;<br>ICD-10 code: I09.81, I11.0, I13.0, I13.2, I50x
Hypertension	ICD-9 code: 401.x – 405.x;<br>ICD-10 code: I10, I11.x, I12.x, I13.x, I15.x, I16.x
Left ventricular hypertrophy	ICD-9 code: 425.1, 429.3;<br>ICD-10 code: I42.1, I42.2, I51.7
MI	ICD-9 code: 410.x, 411.0;<br>ICD-10 code: I21.x, I22.x, I23.x
Peripheral vascular disease	ICD-9 code: 440.2x, 440.3x, 440.4, 440.9;<br>ICD-10 code: I70.x, I73.1, I73.9
Stroke	ICD-9 code: 430, 431, 433.x, 434.x;<br>ICD-10 code: I60.x, I61.x, I63.x
Diabetes drugs	Sustained use = at least 2 fills in any 6-month period. Separate variables for: Metformin; sulfonylurea; GLP-1 receptor antagonists; SGLT-2 inhibitors; DPP-4 inhibitors; TZD's
**Demographic and other information**	
Index fiscal year	2014, 2015, 2016, 2017, 2018, 2019, 2020
Age at index	18-39, 40-49, 50-59, ≥60
Race	African-American, White, Other
Sex	Male, female
Marital status	Married vs. other (on/closest to index date).
VA only health insurance vs. VA+private/Other	- Insurance status variable indicating any access to non-VA health insurance. Coverage defined by most frequently occurring coverage in 2-years prior to index date
Region	Home state geographic region at index. VHA provides home state, which was further categorized based on census region: northeast, Midwest (north central), south, west.
High health services utilization	- Controls for detection bias related to more healthcare encounters.<br>- Average number of outpatient clinic visits per month, calculated as total visits divided by number of months. Total visits is total number of visits in time period of interest. Number of months followed is months from first visit to last visit in time period of interest. Time period of interest is the two years prior to index. The

© 2024 Scherrer JF et al. *JAMA Network Open.*

	distribution of the mean is then dichotomized into high utilizer, >75th percentile vs low utilizer, ≤75th percentile.

© 2024 Scherrer JF et al. *JAMA Network Open.*

**eTable 2.** Age Subgroup Analyses<br>Results from competing risk survival and Cox proportional hazard models estimating the association between PTSD status and type 2 diabetes outcomes, after entropy balance weighting to control for confounding, stratified by age 18-49 vs. 50-80 years of age (n=10,002)		
Outcome (weighted models)	Meets PTSD criteria HR (95% CI)	No longer meets PTSD criteria HR (95% CI)
Starting insulin[a]		
Age 18-49	1.00	0.69 (0.53-0.88)
Age 50-80	1.00	1.11 (0.92-1.33)
*p-value age*Meet PTSD criteria*	*P=.003*	
Poor glycemic control[a]		
Age 18-49	1.00	0.92 (0.81-1.04)
Age 50-80	1.00	1.00 (0.92-1.10)
*p-value age*Meet PTSD criteria*	*P=.26*	
Any microvascular complication[a]		
Age 18-49	1.00	0.90 (0.75-1.07)
Age 50-80	1.00	0.94 (0.86-1.02)
*p-value age*Meet PTSD criteria*	*P=.65*	
Mortality[b]		
Age 18-49	1.00	0.39 (0.19-0.83)
Age 50-80	1.00	1.13 (0.90-1.43)
*p-value age*Meet PTSD criteria*	*P=.008*	

[a] Competing risk survival models
[b] Cox proportional hazard model

© 2024 Scherrer JF et al. *JAMA Network Open.*

eTable 3. Gender Subgroup Analyses
Results from competing risk survival and Cox proportional hazard models estimating the association between PTSD status and type 2 diabetes outcomes, after entropy balancing weighting to control for confounding, stratified by gender (n=10002)

Outcome (weighted models)	Meets PTSD criteria HR (95% CI)	No longer meets PTSD criteria HR (95% CI)
Starting insulin[a]		
Female	1.00	0.65 (0.42-1.01)
Male	1.00	0.97 (0.83-1.13)
*p-value gender*Meet PTSD criteria*	*P=.09*	
Poor glycemic control[a]		
Female	1.00	0.87 (0.70-1.09)
Male	1.00	1.01 (0.93-1.09)
*p-value gender*Meet PTSD criteria*	*P=.24*	
Any microvascular complication[a]		
Female	1.00	0.86 (0.68-1.09)
Male	1.00	0.93 (0.86-1.01)
*p-value gender*Meet PTSD criteria*	*P=.54*	
Mortality[b]		
Female	1.00	0.84 (0.31-2.32)
Male	1.00	1.01 (0.80-1.27)
*p-value gender*Meet PTSD criteria*	*P=.74*	

[a] Competing risk survival models
[b] Cox proportional hazard model

© 2024 Scherrer JF et al. *JAMA Network Open.*

eTable 4. Race Subgroup Analyses

Results from competing risk survival and Cox proportional hazard models estimating the association between PTSD status and type 2 diabetes outcomes, after entropy balance weighting to control for confounding, stratified by race (n=9429)

Outcome (weighted models)	Meets PTSD criteria HR (95% CI)	No longer meets PTSD criteria HR (95% CI)
Starting insulin[a]		
African-American	1.00	0.88 (0.65-1.19)
White	1.00	0.97 (0.82-1.15)
*p-value race*Meet PTSD criteria*	*P=.58*	
Poor glycemic control[a]		
African-American	1.00	0.93 (0.80-1.09)
White	1.00	1.03 (0.95-1.13)
*p-value race*Meet PTSD criteria*	*P=.26*	
Any microvascular complication[a]		
African-American	1.00	0.98 (0.84-1.14)
White	1.00	0.90 (0.82-0.98)
*p-value race*Meet PTSD criteria*	*P=.33*	
Mortality[b]		
African-American	1.00	1.31 (0.82-2.09)
White	1.00	0.90 (0.70-1.17)
*p-value race*Meet PTSD criteria*	*P=.17*	

[a] Competing risk survival models
[b] Cox proportional hazard model

eTable 5. Depression Subgroup Analyses

Results from competing risk survival and Cox proportional hazard models estimating the association between PTSD status and type 2 diabetes outcomes, after entropy balance weighting to control for confounding, stratified by depression status (n=10002)

Outcome (weighted models)	Meets PTSD criteria HR (95% CI)	No longer meets PTSD criteria HR (95% CI)
Starting insulin[a]		
No depression	1.00	0.73 (0.55-0.97)
Depression	1.00	1.04 (0.88-1.24)
*p-value depression*Meet PTSD criteria*	*P=.03*	
Poor glycemic control[a]		
No depression	1.00	0.95 (0.84-1.07)
Depression	1.00	1.03 (0.94-1.13)
*p-value depression*Meet PTSD criteria*	*P=.30*	
Any microvascular complication[a]		
No depression	1.00	0.96 (0.84-1.09)
Depression	1.00	0.91 (0.82-0.99)
*p-value depression*Meet PTSD criteria*	*P=.53*	
Mortality[b]		
No depression	1.00	0.96 (0.65-1.44)
Depression	1.00	1.01 (0.77-1.33)
*p-value depression*Meet PTSD criteria*	*P=.84*	

[a] Competing risk survival models
[b] Cox proportional hazard model

© 2024 Scherrer JF et al. *JAMA Network Open.*

eTable 6. Posttraumatic Stress Disorder (PTSD) Severity Subgroup Analyses

Competing risk survival and Cox proportional hazard models estimating the association between PTSD status and type 2 diabetes outcomes after entropy balance weighting to control for confounding stratified by PTSD severity (PCL score 1 ≥66 vs < 66) (n=10002)

Outcome (weighted models)	Meets PTSD criteria HR (95% CI)	No longer meets PTSD criteria HR (95% CI)
Starting insulin[a]		
PTSD Not severe	1.00	0.99 (0.85-1.16)
PTSD Severe	1.00	0.63 (0.38-1.07)
*p-value severity*Meet PTSD criteria*	*P=.11*	
Poor glycemic control[a]		
PTSD Not severe	1.00	1.00 (0.92-1.08)
PTSD Severe	1.00	1.03 (0.82-1.30)
*p-value severity*Meet PTSD criteria*	*P=.80*	
Any microvascular complication[a]		
PTSD Not severe	1.00	0.96 (0.89-1.04)
PTSD Severe	1.00	0.85 (0.65-1.11)
*p-value severity*Meet PTSD criteria*	*P=.38*	
Mortality[b]		
PTSD Not severe	1.00	0.96 (0.77-1.20)
PTSD Severe	1.00	1.19 (0.56-2.54)
*p-value severity*Meet PTSD criteria*	*P=.59*	

[a] Competing risk survival models
[b] Cox proportional hazard model

© 2024 Scherrer JF et al. *JAMA Network Open.*

eTable 7. Four Levels of PTSD Checklist (PCL) Severity Groups

Last PCL in exposure year (index PCL) groups by outcomes (severity of last PCL in exposure year and relationship to outcomes). Fully adjusted hazard ratios and 95% confidence intervals.

Outcome	Index PCL ≤18 (n=728)	Index PCL 19-32 (n=1396)	Index PCL 33-65 (n=6400)	Index PCL 66-80 (n=1478)	p-value
Starting insulin					
Incidence rate per 1000PY	20.8/1000PY	23.4/1000PY	24.1/1000PY	25.8/1000PY	.459
Hazard ratio (CI)	1.00	1.07 (0.81-1.43)	1.10 (0.86-1.41)	1.10 (0.83-1.47)	---
Poor glycemic control					
Incidence rate per 1000PY	135.9/1000PY	137.7/1000PY	135.2/1000PY	127.4/1000PY	.513
[a]Hazard ratio (CI)	1.00	0.97 (0.84-1.11)	0.97 (0.86-1.10)	0.91 (0.79-1.05)	---
Any microvascular complication					
Incidence rate per 1000PY	98.0/1000PY	114.1/1000PY	107.2/1000PY	95.3/1000PY	.010
[a]Hazard ratio (CI)	1.00	1.23 (1.06-1.42)	1.26 (1.11-1.43)	1.24 (1.06-1.44)	---
Mortality					
Incidence rate per 1000PY	12.8/1000PY	10.4/1000PY	11.2/1000PY	9.9/1000PY	.492
Hazard ratio (CI)	1.00	0.90 (0.62-1.30)	0.99 (0.73-1.34)	0.87 (0.59-1.27)	---

© 2024 Scherrer JF et al. *JAMA Network Open.*
