# Supplementary material for: Posttraumatic Stress Disorder and Type 2 Diabetes Outcomes in Veterans
Source: JAMA Netw Open. 2024 Aug 13;7(8):e2427569. doi: 10.1001/jamanetworkopen.2024.27569 (PMC11322846; doi:10.1001/jamanetworkopen.2024.27569)
Supplement: Supplement 2. — Data Sharing Statement [file jamanetwopen-e2427569-s002.pdf]

Data Sharing Statement

Scherrer. Posttraumatic Stress Disorder and Type 2 Diabetes Outcomes in Veterans. *JAMA Netw Open*. Published August 13, 2024. doi:10.1001/jamanetworkopen.2024.27569

Data
Data available: Yes
Data types: Deidentified participant data
How to access data: Persons who obtain VA affiliation, IRB approval and a DUA. Interested parties can contact the corresponding author
When available: With publication

Supporting Documents
Document types: Statistical/analytic code
How to access documents: Made available by contacting jeffrey.scherrer@health.slu.edu
When available: With publication

Additional Information
Who can access the data: appropriate requests from qualified investigators
Types of analyses: PTSD
Mechanisms of data availability: DUA
Any additional restrictions: VA IRB
